# Supplementary material for: Age Structure, Development and Population Viability of Banteng (Bos javanicus) in Captive Breeding for Ex-Situ Conservation and Reintroduction
Source: Animals (Basel). 2023 Jan 5;13(2):198. doi: 10.3390/ani13020198 (PMC9854904; doi:10.3390/ani13020198)
Supplement: Supplementary file 1 [file animals-13-00198-s001.zip › animals-2066762-supplementary.pdf]

Article

Age Structure, Development and Population Viability of Banteng (*Bos javanicus*) in Captive Breeding for Ex-Situ Conservation and Reintroduction

Rattanawat Chaiyarat [1,*], Neeracha Sriphonkrang [1], Phattaranan Khamsirinan [1], Saree Nakbun [2] and Namphung Youngpoy [1]

[1] Wildlife and Plant Research Center, Faculty of Environment and Resource Studies, Mahidol University, Nakhon Pathom 73170, Thailand

[2] Khao Nampu Nature and Wildlife Education Center, Department of National Parks, Wildlife and Plant Conservation, Kanchanaburi 71250, Thailand

* Correspondence: rattanawat.cha@mahidol.ac.th

Supplementary Materials

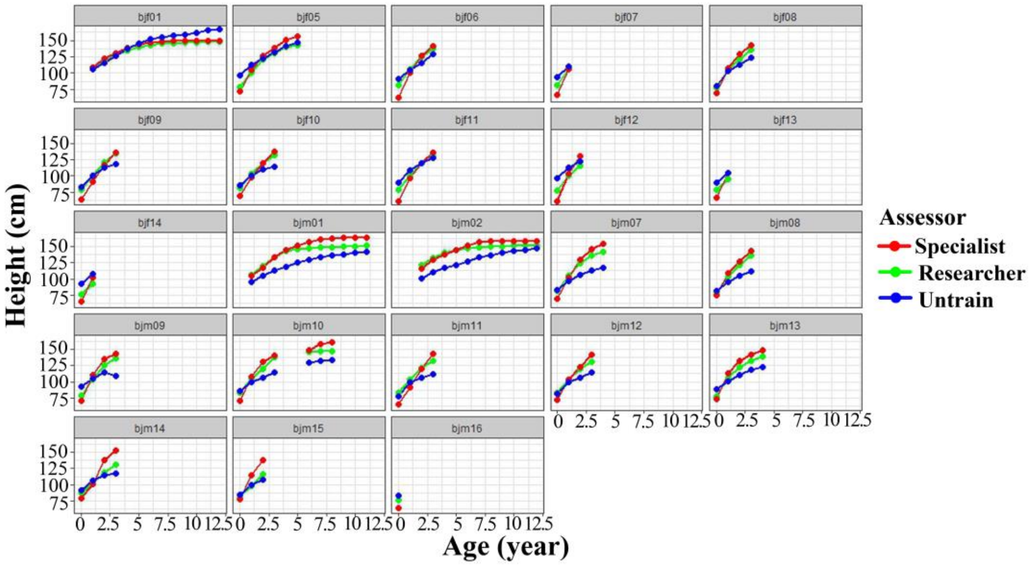

Figure S1. Relationship between age and height of individual captive banteng from three assessor groups: bjm = male *Bos javanicus*; bjf = female *Bos javanicus*.

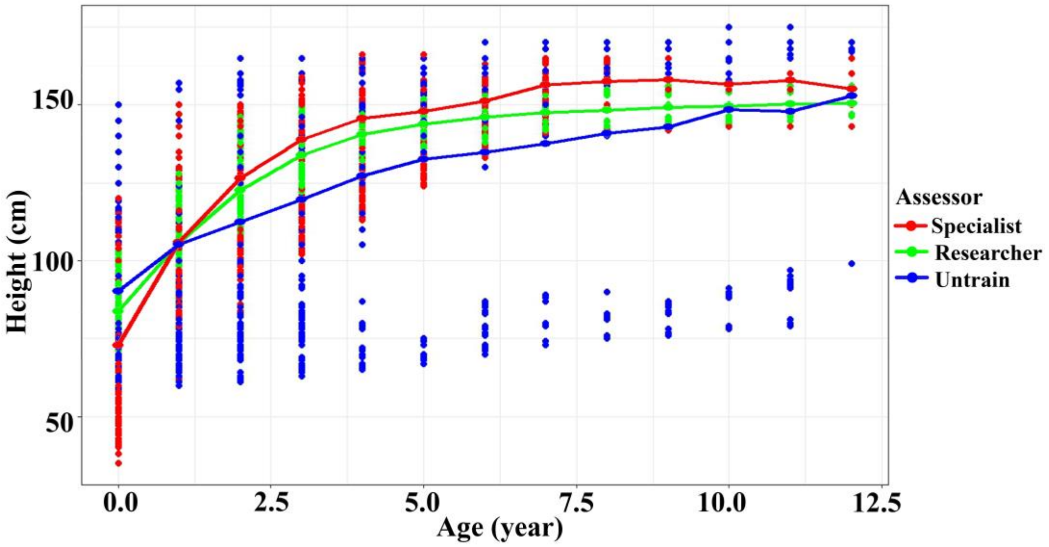

Figure S2. Relationship between age and height of captive banteng from three assessor groups.

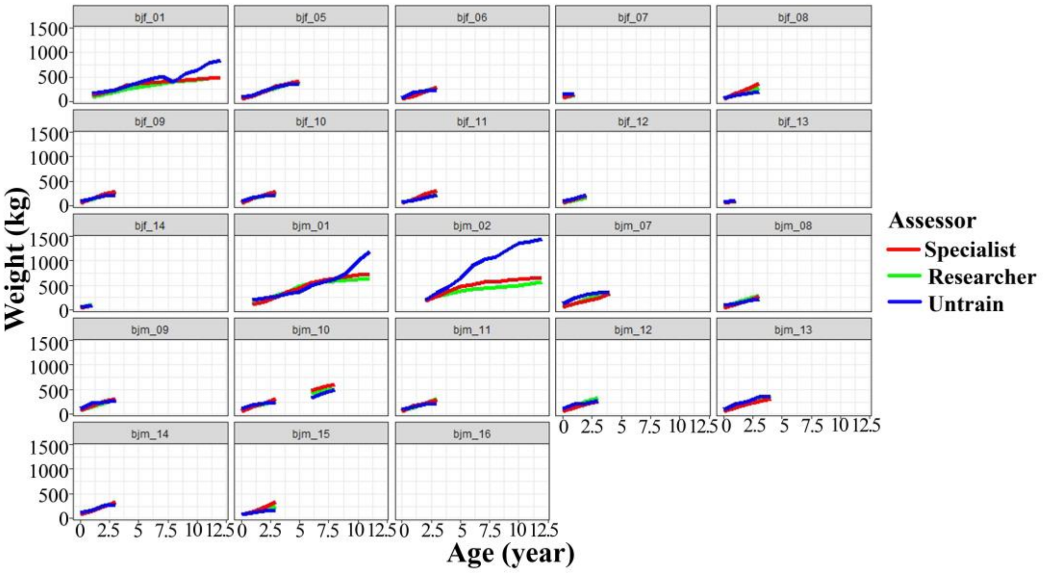

Figure S3. Relationship between age and weight of individual captive banteng from three assessor groups: bjm = male *Bos javanicus*; bjf = female *Bos javanicus*.

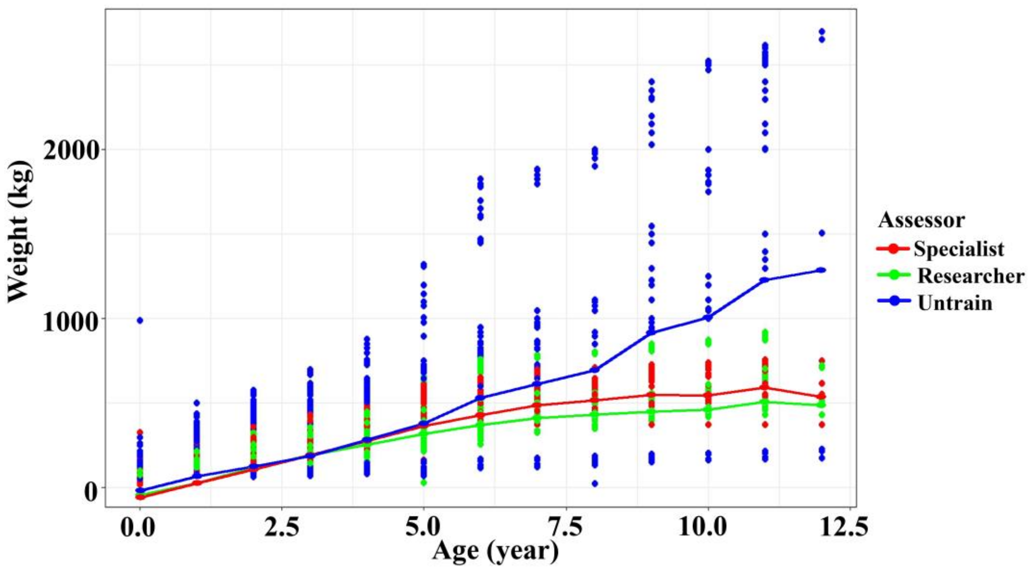

Figure S4. Relationship between age and weight of (a) male and (b) female captive banteng by researchers.

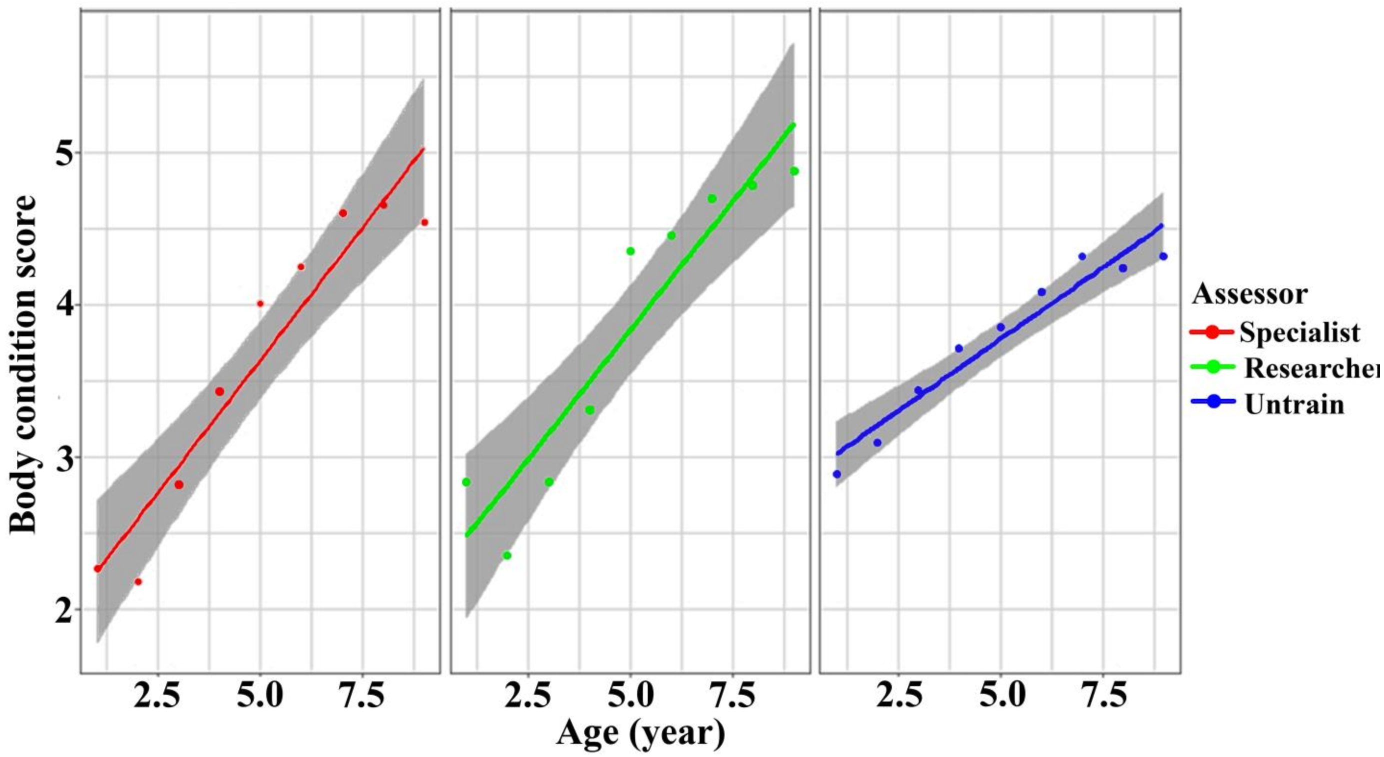

(a) (b) (c)

Figure S5. Relationship between age and body condition score by (a) specialists (b) researchers and (c) untrain.

Table S1. Relationship between age (*y*), height (*h*), weight (*w*) and body condition score (*b*) of captive banteng.

Cattle Sex	Assessor	Model	R^2	*p*-value
Both	Specialist	$yh = 163.33 + 0.04 \times 0.40\ln(x)$	0.29	<0.001
Both	Researcher	$yh = 151.5 + 0.23 \times 0.26\ln(x)$	0.41	<0.001

Both	Untrain	$yh = 167.67 + 0.23 \times 0.20\ln(x)$	0.08	0.023
Male	Researcher	$yh = 151.50 + 0.28 \times 0.26\ln(x)$	0.28	<0.001
Female	Researcher	$yh = 148.50 + 0.19 \times 0.26\ln(x)$	0.30	0.001
Both	Specialist	$yh = 728.9 - 2.3 \times 0.21\ln(x)$	0.004	0.68
Both	Researcher	$yw = 634.24 - 2.27 \times 0.18\ln(x)$	0.056	0.16
Both	Untrain	$yw = 1449.5 - 2.4 \times 0.31\ln(x)$	0.48	<0.001
Male	Researcher	$yw = 634 - 2.16 \times 0.19\ln(x)$	-0.06	0.236
Female	Researcher	$yw = 483 - 2.42 \times 0.17\ln(x)$	-0.04	0.48
Both	Specialist	$yb = 1.40 + 0.42x$	0.71	<0.001
Both	Researcher	$yb = 1.80 + 0.58x$	0.69	<0.001
Both	Untrain	$yb = 2.51 + 0.24x$	0.59	<0.001
Male	Researcher	$yb = 2.32 + 0.31x$	0.78	<0.001
Female	Researcher	$yb = 2.35 + 0.23x$	0.70	<0.001
